# Human endogenous retrovirus HERV-K(HML-2) encodes a stable signal peptide with biological properties distinct from Rec

**DOI:** 10.1186/1742-4690-6-17

**Published:** 2009-02-16

**Authors:** Alessia Ruggieri, Esther Maldener, Marlies Sauter, Nikolaus Mueller-Lantzsch, Eckart Meese, Oliver T Fackler, Jens Mayer

**Affiliations:** 1 Department of Human Genetics, Medical Faculty, University of Saarland, Homburg, Germany; 2 Institute of Virology, Medical Faculty, University of Saarland, Homburg, Germany; 3 Department of Virology, University of Heidelberg, Heidelberg, Germany; 4 Department of Molecular Virology, Im Neuenheimer Feld 345, University of Heidelberg, 69120 Heidelberg, Germany

## Abstract

**Background:**

The human endogenous retrovirus HERV-K(HML-2) family is associated with testicular germ cell tumors (GCT). Various HML-2 proviruses encode viral proteins such as Env and Rec.

**Results:**

We describe here that HML-2 Env gives rise to a 13 kDa signal peptide (SP) that harbors a different C-terminus compared to Rec. Subsequent to guiding Env to the endoplasmatic reticulum (ER), HML-2 SP is released into the cytosol. Biochemical analysis and confocal microscopy demonstrated that similar to Rec, SP efficiently translocates to the granular component of nucleoli. Unlike Rec, SP does not shuttle between nucleus and cytoplasm. SP is less stable than Rec as it is subjected to proteasomal degradation. Moreover, SP lacks export activity towards HML-2 genomic RNA, the main function of Rec in the original viral context, and SP does not interfere with Rec's RNA export activity.

**Conclusion:**

SP is a previously unrecognized HML-2 protein that, besides targeting and translocation of Env into the ER lumen, may exert biological functions distinct from Rec. HML-2 SP represents another functional similarity with the closely related Mouse Mammary Tumor Virus that encodes an Env-derived SP named p14. Our findings furthermore support the emerging concept of bioactive SPs as a conserved retroviral strategy to modulate their host cell environment, evidenced here by a "retroviral fossil". While the specific role of HML-2 SP remains to be elucidated in the context of human biology, we speculate that it may be involved in immune evasion of GCT cells or tumorigenesis.

## Background

The human genome harbors about 8% of sequences of retroviral origin, remnants of different exogenous retrovirus infections of the germ line genome that occurred millions of years ago. The human endogenous retrovirus (HERV) family HERV-K(HML-2), henceforth HML-2, family contains recently formed proviral loci. The number of mutations along the proviral coding sequence remains low for evolutionarily younger HML-2 proviral loci. Some of those proviruses contain nearly intact open reading frames (ORFs) with a few or no mutations [1-4] and functional proteins *in vitro *[5-11]. Though, while engineered HML-2 proviruses display *ex vivo *infectivity and ability to form new proviruses [12,13], no replication-competent HERV-K(HML-2) variant was identified in the human population so far. The HML-2 family was also shown to produce retrovirus-like particles budding from teratocarcinoma and melanoma derived cell lines [14,15]. HERVs have been implicated in several human pathologies including cancers and autoimmune diseases [reviewed in [16,17]]. HML-2 has gained special attention because of its association with testicular germ cell tumors (GCT), the most common tumor type among young men in western industrialized countries. Indeed, HML-2 expression is strongly up-regulated in early stages of GCT [18]. Eighty-five percent of GCT patients, more precisely seminoma patients, display a specific immune response to HML-2 Gag and Env proteins [19,20]. Since tumor remissions are associated with a decreased titer, while progression or relapse coincide with stable or elevated titers, antibody titers correlate with clinical manifestation of the disease [21,22].

Two major types of HML-2 proviruses exist in the genome. Type 1 proviruses differ from full-length type 2 proviruses by a 292 bp deletion within the boundary of *pol *and *env *genes [23,24]*env *mRNA from type 2 proviruses is subspliced to create a *rec *mRNA that encodes the Rec (formerly cORF) protein, a functional homologue to Rev and Rex, the RNA-binding nuclear export proteins of HIV and HTLV, respectively [25-29]. Rec has been reported to interact with nuclear promyelocytic leukemia zinc finger (PLZF) protein that has been implicated in leukemogenesis and spermatogenesis, and disturbs germ cell development in Rec-transgenic mice [30-32]. Type 1 sequences lack the *rec *splice donor site that is located in the 292 bp stretch [27]. An alternative splice donor site located just upstream of the 292 bp stretch is instead used to splice *np9 *mRNA. The corresponding Np9 protein shares only 14 aa with Rec and Env [33,34].

HERV-K(HML-2) displays significant sequence similarities with Mouse Mammary Tumor virus (MMTV), particularly for the *env *gene [35]. Both HML-2 and MMTV belong to the *Betaretroviruses *that include retroviruses formerly classified as type B and D [36]. MMTV also encodes a functional homologue of HIV Rev and HML-2 Rec, termed Rem [37,38]. Rem contains the complete and unusually long signal peptide of MMTV Env precursor, termed of p14/SP_Rem_. The latter was shown to translocate into nucleoli of murine T cell lymphoma cells [39,40]. Specific functions of p14/SP_Rem _remain to be elucidated.

Characterization of presecretory eukaryotic and prokaryotic signal peptides (SPs) defined the features essential for their function, such as hydrophobicity and a common sequence for the site of cleavage from its mature protein by signal peptidase [41-43]. For many cellular proteins, SP's unique function is to target nascent polypeptide chains into the endoplasmic reticulum (ER) membrane and entry into the translocon. While much is known about subsequent transport of the secretory protein to its correct subcellular location, the fate of signal peptides after their cleavage from the pre-proteins is still unclear and turns out to be complex. SP degradation kinetic and longevity are variable. In some cases, SPs are thought to be readily degraded, making them undetectable *in vitro*. Some SPs are further processed by an ER intramembrane cleaving protease, the signal peptide peptidase and released into the cytosol where they can accumulate [44-46]. Importantly, according to this emerging concept, these "longer-living" SPs, liberated into the cytosol, could promote post-targeting functions in the cell, such as cell signaling or regulation [47].

The orientation of SPs across the ER membrane defines two types of signal peptides. Type I SPs anchor the proteins by transferring it across the ER membrane, leaving the C-terminus of the protein in the cytoplasmic side of the ER. Conversely, type II SPs retain the N-terminus of the protein in the cytosol [45,48]. Retroviral Env SPs are type II membrane proteins. In most cases, after polypeptide chain transfer into the translocon, SP is cleaved from the Env precursor by signal peptidase and subsequently degraded. Env monomers integrate into the ER membrane and undergo further maturation steps [49,50]. However, besides MMTV p14/SP_Rem_, several exceptions exist: HIV-1 gp120 Env SP remains bound to calnexin in the ER membrane and is inefficiently cleaved very late in the maturation process [51,52]. For Human Foamy Virus (HFV), SP mediates specificity of Env interaction with HFV capsid and is found in purified particles [53].

More recently, biochemical studies showed that p14/SP_Rem _targets Rem to the ER, is then cleaved off and accumulates in the nucleoli. Interestingly, this process is independent of cleavage by signal peptide peptidase [54].

We describe here for the first time the HERV-K(HML2) Env precursor SP as a 13 kDa signal peptide. By examining features of HML-2 SP, such as subcellular localization, nucleocytoplasmic shuttling, protein stability and RNA export activity, we established functional dissimilarities to Rec. Our data suggest that HML-2 SP exerts a Rec-independent function. Furthermore, the finding of a long-lived SP for HML-2 reveals another similarity between the closely related HML-2 and MMTV retroviruses, thus further establishes their close relationship on the functional level.

## Results

### SPs among the Retroviridae

**Figure 1:**
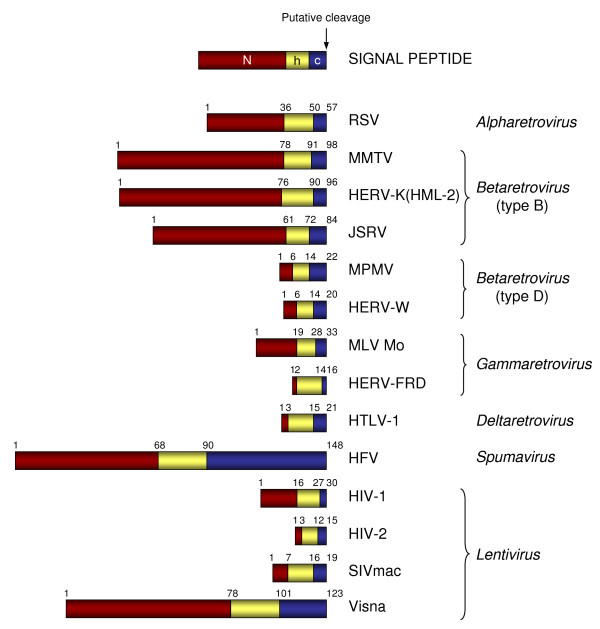
**Domain organization of SPs of selected retroviruses**. The tripartite composition of retroviral SPs was analyzed using PHOBIUS [55], SignalP [56] and TMD [57]. Characteristic domains in representative exogenous and endogenous prototypes of each *Retroviridae *class are shown. Betaretroviruses are further classified based on an earlier retrovirus taxonomy. See text for details on N-terminal extremity (N); central hydrophobic core (h); C-terminal extremity (c). Numbers indicate start and end positions, in aa, of each domain. RSV: Rous Sarcoma Virus; MMTV: Mouse Mammary Tumor Virus; HERV-K(HML-2): Human Endogenous Retrovirus type K subfamily HML-2; JSRV: Jaagsiekte Sheep Retrovirus; MPMV: Mason Pfizer Monkey Retrovirus; HERV-W: Human Endogenous Retrovirus type W; MLV Mo: Moloney Murine Leukemia Virus; HERV-FRD: Human Endogenous Retrovirus type FRD; HTLV-1: Human T-cell Leukemia Virus 1; HFV: Human Foamy Virus. HIV-1/HIV-2: Human Immunodeficiency Virus 1 and 2; SIVmac: Simian Immunodeficiency Virus, acaque isolate; Visna: Maedi-Visna Virus.

To gain better insight into the organization of retroviral SPs, we first compared the SP regions of prototype members of each *Retroviridae *class and related endogenous retroviral members, using PHOBIUS, SignalP and TMD [55-57]. As depicted in Figure 1, Retroviridae SPs vary significantly in length, with the shortest one being the 15 aa long HIV-2 SP and the longest one being the 148 aa long HFV SP. Diverse prototypes of Lentiviruses, including primate and ungulate Lentiviruses, underline that such heterogeneity in SP length also exists among different members of the same class. All SPs analyzed share a characteristic tripartite composition [58]. The central hydrophobic core (h), critical for targeting and insertion into the ER membrane [42], encompasses between 11 and 22 residues. The C-terminal extremity (c) is a small polar region that determines the signal peptidase cleavage site and is well conserved among all retroviruses analyzed, with the exception of the HFV prototype. Of note, the N-terminal extremity (N), which is not involved in protein insertion and translocation, is very little conserved in amino acid sequence and length [59]. For HERV-K(HML-2), as well as for the other *Betaretrovirus *prototypes, SP N-extensions consist of an unusually long sequence varying from 61 to 78 residues.

### HML-2 SP sequence motifs

**Figure 2:**
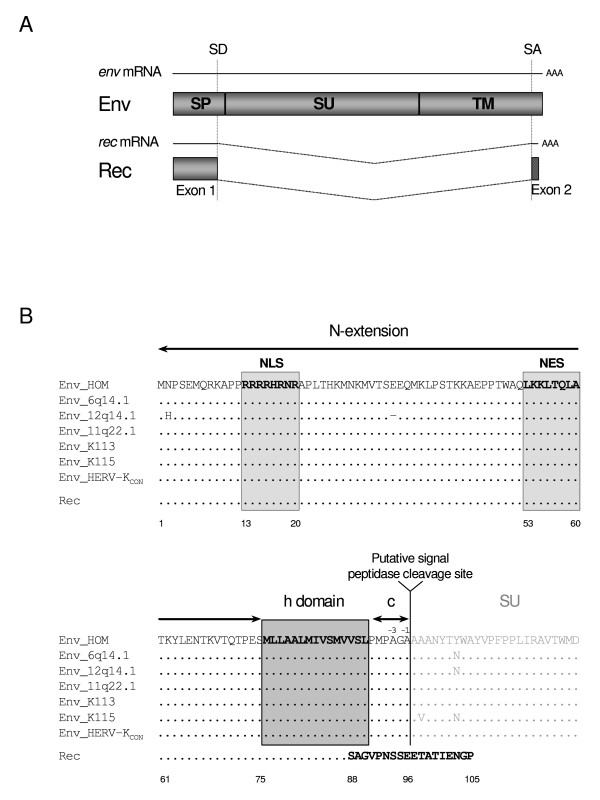
**Comparison of HERV-K(HML-2) SP and Rec sequences**. (A) *env *mRNA encodes an Env precursor protein that is cleaved in the ER by signal peptidase releasing SP. In the Golgi, the Env precursor is further processed and cleaved by a furin-like endoprotease to give rise to surface (SU) and transmembrane (TM) subunits. *rec *mRNA is a splice product of *env *mRNA and encodes Rec. The first exon of Rec overlaps with SP while the second exon is translated from a different reading frame. SD/SA: *rec *splice donor and acceptor sites. (B) SP and Rec amino acid sequence alignment. The human genome contains six proviruses with complete Env ORFs [13]. HERV-K(HML-2.HOM) is an almost intact provirus located on chromosome 7p22.1 (Env_HOM) [60]. Chromosomal localizations of other Env encoding loci are indicated. HERV-K113 (Env_K113) and HERV-K115 (Env_K115) are two polymorphic proviruses located on chromosomes 19p12 and 8p23.1, respectively [4]. The alignment also includes HERV-K_CON_, a recently engineered "infectious" provirus [12], and the Rec sequence [27]. Rec exon 1 (aa 1–87) is also found in SP while the second exon of Rec (aa 88–105) is translated from a different reading frame. N: N-extension (aa 1–75); h: hydrophobic h domain (aa 76–90); C: polar domain (aa 91–96); -3,-1:position of small uncharged residues. By analogy with motifs previously characterized in Rec, a putative arginine-rich nuclear localization signal (NLS; aa 13–20) and a leucine-rich nuclear export signal (NES; aa 54–60) are present in HML-2 SP.

HERV-K(HML-2) Env is synthesized as a classical retroviral envelope protein. In the ER, the Env precursor undergoes a first cleavage by the signal peptidase releasing the 90 kDa Env precursor which then follows the maturation pathway to the Golgi where it is further cleaved by a furin-like endoprotease into two N-glycosylated domains, a 55 kDa surface subunit (SU) and a 39 kDa transmembrane subunit (TM) (A. Ruggieri, unpublished data). In addition to SU and TM, an accessory protein Rec is encoded by a smaller mRNA resulting from *env *mRNA subsplicing. The first exon of Rec largely overlaps with the *env *SP coding sequence in that it comprises amino acids 1 to 87 of Env. The second exon of Rec is translated from a different reading frame. The resulting 18aa C-terminus is different in sequence from either the C-terminus of SP or Env. With regard to the resulting protein, Rec mRNA splicing occurs just upstream of the SPase cleavage site (Figure 2A). Contrary to MMTV Rem, Rec does not contain the complete SP sequence.

In order to determine conservation of SP among HML-2 proviruses and its sequence relationship to Rec, we compared relevant sequence portions of six HML-2 loci that could potentially encode full-length Env [13], the sequence of recently engineered HML-2 Envs, HERV-K_CON_/Phoenix [12,13], representative of a functional and "infectious" HML-2 Env, and the Rec sequence as previously reported [27] (Figure 2B). The sequences were almost identical with each other, with complete identity between HERV-K(HML-2.HOM), an almost intact HML-2 provirus located on chromosome 7 [60], and the "infectious" HERV-K_CON _[12]. Comparison of the 96 aa long SP with the 105 aa long Rec showed that both proteins share the identical N-terminal 87 aa, whereas the C-terminal 9 and 18 aa for SP and Rec, respectively, are unrelated in sequence (Figure 2B) for reasons described above. By analogy with previously characterized Rec [27,61], HML-2 SP harbors two conserved motifs: an arginine-rich putative nuclear localization signal (NLS; aa 13–20) and a leucine-rich putative nuclear export signal (NES; aa 54–60). Additionally, HML-2 SP contains domains characteristic for cellular SPs: (i) a positively charged long N-extension (residues 1–75), (ii) a hydrophobic h domain (residues 76–90) and (iii) a short polar domain (residues 91–96) containing characteristic helix-breaking proline and glycine residues as well as small uncharged residues in position -3 and -1 adjacent to the h domain [58,62]. HML-2 SP therefore displays a tripartite structure characteristic of SPs and contains an unusually long N-extension bearing putative trafficking motifs. The similarities between HML-2 SP and Rec proteins, in terms of length and sequence, prompted us to investigate functional similarities and differences between the two proteins.

### HML-2 SP-RFP fusion proteins can localize in nucleoli

**Figure 3:**
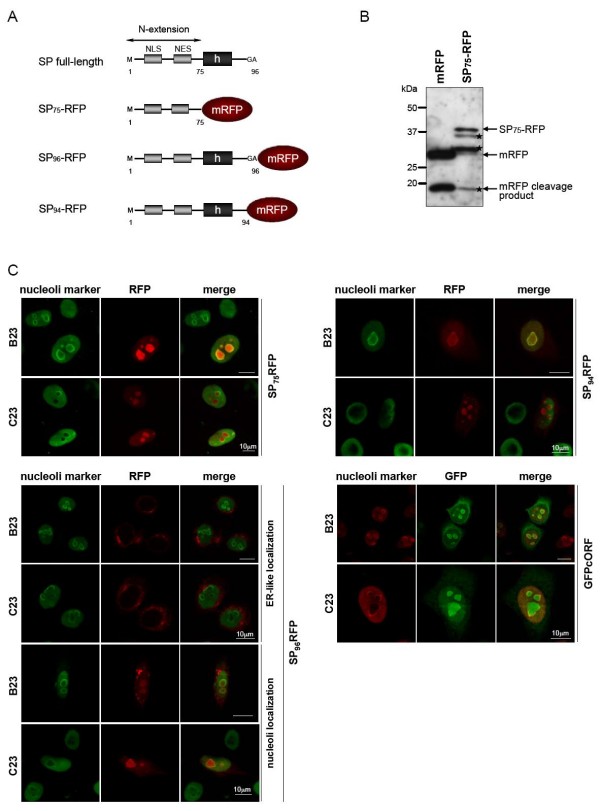
**Subcellular localization of HML-2 SP fusion protein**. (A) Schematic representation of SP fusion proteins. N-extensions of HML-2 SP of different length (aa 1–75; aa 1–94; aa 1–96) were cloned in frame with the monomeric red fluorescent protein (mRFP). NLS: putative nuclear localization signal; NES: putative nuclear export signal; h: hydrophobic core. (B) Western blot analysis of HeLa cells transiently expressing mRFP and SP_75_-RFP. The Western blot was stained with anti-mRFP antibody. mRFP-expressing cells produce mRFP with an approximate molecular weight of 30 kDa. An 18–19 kDa proteolytic product can also be is detected. An SP_75_-RFP construct produces SP-RFP fusion protein and (RFP) degradation products. (C) Confocal sections showing SP_75_-RFP, SP_96_-RFP and SP_94_-RFP fluorescence in red and co-immunostained nucleolar markers B23/nucleophosmin or C23/nucleolin in green. The lower right panel shows GFPcORF/Rec fluorescence in green and nucleolar markers in red. Co-localization of proteins is indicated in yellow. Images show HeLa cells fixed 24 hours post-transfection. White bar = 10 μm.

**Figure 4:**
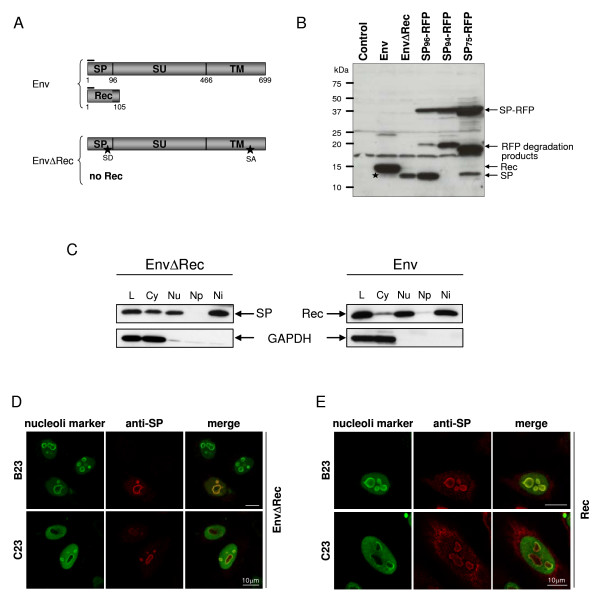
**HERV-K(HML-2) SP localizes to nucleoli**. (A) Schematic representation of proteins encoded by Env and EnvΔRec expression vectors. Env-expressing cells produce Env and Rec. Both contain the epitope recognized by the anti-SP antibody (black bars). The EnvΔRec construct harbors silent point mutations (asterisks) in splice donor (SD) and splice acceptor (SA) sites, eliminating *rec *mRNA splicing and Rec protein production. (B) Western blot analysis of HeLa cells transiently expressing Env, EnvΔRec and SP_96/94/75_-RFP. The Western blot was stained with anti-SP antibody. Env-expressing cells produce the 15 kDa Rec protein and a lower amount of SP (asterisk) with an approximate molecular weight of 13 kDa. In EnvΔRec-expressing cells only SP is detected. SP_96/94/75_-RFP constructs produce SP-RFP fusion proteins and (RFP) degradation products. SP_96_-RFP releases SP while SP_75_-RFP and SP_94_-RFP do not due to engineered deletions (see text). An SP-like band produced by SP_75_-RFP is very likely an unspecific mRFP degradation product (see text and Figure 3B). (C) Western blot analysis of fractionated Hela cells transiently expressing Env and EnvΔRec. Cell lysates were probed with anti-SP and anti-GAPDH antibodies, the latter verifying proper separation of fractions. L: full lysate; Cy: cytoplasm; Nu: nucleus; Np: nucleoplasm; Ni: nucleoli. (D and E) Confocal sections of HeLa cells fixed 24 hours post-transfection and co-immunostained with anti-SP for detection of SP or Rec (in red), and with antibodies detecting B23/nucleophosmin or C23/nucleolin (in green). White bar = 10 μm. (D) SP distribution in EnvΔRec-expressing cells. (E) Rec distribution in Env-expressing cells. The merge panels show, in yellow, co-localization of both SP and Rec with B23/nucleophosmin in the granular component of nucleoli.

We first determined the subcellular localization of SP and Rec. To this end, three SP expression constructs where generated by cloning SP sequences of variable length upstream of the *mrfp *gene coding for monomeric Red Fluorescent Protein (mRFP) (Figure 3A). The first construct, named SP_75_-RFP, included only the HML-2 SP N-extension sequence (aa 1–75) that bears the NLS and NES and that is common with Rec. SP_96_-RFP (aa 1–96) corresponded to the full-length HML-2 SP sequence. To prevent or diminish signal peptidase cleavage during synthesis of SP_96_-RFP, we deleted the C-terminal two aa residues (GA) of the HML-2 SP, giving rise to construct SP_94_-RFP. Deletion of those two aa was based on a consensus sequence for signal peptidase cleavage, in which small uncharged residues in position -3 and -1, including a glycine residue, are thought to be important for cleavage [62]. Proper protein expression from SP_96/94/75_-RFP precursor proteins was verified by Western Blot (Figures 3B and 4B). Figure 3B shows a Western Blot analysis of HeLa cells expressing for 48 hours either mRFP or SP_75_-RFP, probed with an anti-mRFP polyclonal antibody. The mRFP protein is subjected to proteolytical degradation, as indicated by an 18-19 kDa band. Probing SP fusion contructs with an anti-SP polyclonal antibody confirmed the absence of cleaved SP in the SP_94_-RFP construct, in which the signal peptidase cleavage site had been mutated. As observed in control experiments (Figure 3B), mRFP fusion protein is likely proteotically cleaved in the mRFP moiety or in the linker region between mRFP and SP sequences. Unspecific cleavage likely occured in this linker region for SP_75_-RFP and SP_94_-RFP proteins, giving rise to bands with noticeably different sizes (Figure 4B).

We compared the subcellular localization of these three SP-RFP fusion contructs to that of the previously described GFPcORF, a biologically active Rec fused to Green Fluoresccent Protein (GFP) [61]. HeLa cells were transiently transfected, fixed after 24 hours and analyzed by confocal microscopy. HeLa cells expressing either mRFP or eGFP were transfected and analyzed as control. Both mRFP and eGFP were homogeneously distributed in the cell (data not shown). As expected for a protein shuttling between cytoplasm and nucleoli, GFPcORF was found in both cytoplasm and nucleoli, as confirmed by counterstaining of cells with antibodies against B23/nucleophosmin or C23/nucleolin, two major proteins of the granular component and the dense fibrillar component of the nucleolar compartment [63]. SP_75_-RFP was strongly enriched in the nucleoli of transfected cells and could also be detected in the cytoplasm, however, at significantly lower amounts than GFPcORF that was visible only after over-exposure (Figure 3C; and data not shown). These observations revealed that the 75 aa long N-extension domain of HML-2 SP, that is identical to the N-terminal part of Rec, efficiently targets an mRFP fusion moiety to nucleoli resulting in a subcellular distribution similar, but not identical, to that of Rec. The full-length SP fusion protein, SP_96_-RFP, displayed an unexpected phenotype. In about half of the transfected cells, subcellular localization of SP_96_-RFP was suggestive of the ER network, probably indicating that SP_96_-RFP was following the ER pathway and that HML-2 SP was still able to achieve its primary function, namely translocating proteins into the ER membrane. For the remaining transfected cells SP_96_-RFP was found in the nucleoli (Figure 3C). As the cells expressing the different SP fusion constructs were transiently transfected, we hypothesized that the ER pathway was saturated and that for this fraction of cells proteins were translated on free ribosomes in the cytosol. Interestingly, SP_94_-RFP also displayed a subcellular localization identical to that of SP_75_-RFP and GFPcORF, likewise accumulating in nucleoli. The synthesis pathway followed by SP_94_-RFP protein is also difficult to predict as the SP precursor protein lacks a proper signal peptidase cleavage site. SP_75_-RFP and SP_94_-RFP are likely synthesized in the cytosol. Taken together, our results confirm that HML-2 SP sequence is capable of translocating a precursor protein to the ER and can target a cytosolic protein to the nucleoli.

### HML-2 SP accumulates in the granular component of nucleoli

After cleavage from the native polypeptidic chain, SPs are usually released from the ER membrane and subsequently degraded. In some cases and in particular when the N-extension is long, SPs can be released into the cytosol and exert biological activities [45]. As above results suggested that HML-2 SP contains a functional NLS in the N-terminal extension, we addressed whether HML-2 SP localizes to nucleoli when cleaved from the Env precursor protein. To exclude the possibility that the observed localization of SP_94_-RFP, and in some cases that of SP_96_-RFP, was due to an aberrant conformation generated by the C-terminal RFP fusion, we determined HML-2 SP's localization after cleavage from its natural Env precursor. To facilitate analysis of SP in the Env context and to eliminate Rec production, we introduced silent mutations in *rec *splice donor and acceptor sites at nt positions 6708–6716 and 8404–8414, respectively (numbers refer to the HERV-K(HML-2.HOM) sequence [60] (EnvΔRec; Figure 4A). Presence of *env *and *rec *transcripts was analyzed by RT-PCR using appropriate primers. While *env *transcripts were readily observed, no transcript corresponding to *rec *mRNA could be detected (data not shown). Hence, the EnvΔRec construct predominantly produces SP and Env but no Rec. Furthermore, Env maturation and trafficking were not affected by the introduced point mutations, and as predicted, as more *env *mRNA was available for translation, EnvΔRec-expressing cells showed an increased amount of Env (data not shown).

To detect HML-2 SP, we raised a rabbit polyclonal antiserum against the N-terminal 19 aa of HML-2 Env (anti-SP). That anti-SP antibody detected a protein of approx. 15 kDa, as predicted for Rec, in Env-expressing cells (Figure 4B). In EnvΔRec-expressing cells, only SP with a molecular weight of approx. 13 kDa, but not Rec, could be detected. Interestingly, at high levels of wild-type Env expression, besides Rec, another smaller and fainter band corresponding to the size of SP could be detected, indicating that SP is produced at low steady-state levels also from the wild-type HML-2 Env precursor. However, the anti-SP antibody did not allow detection of the Env precursor, likely because of conformational inaccessibility of the recognized epitope.

We used EnvΔRec-expressing cells to determine the subcellular localization of HML-2 SP and compared it to that of Rec. Considering that the epitope designed for anti-SP production is also present in Rec, the antibody thus detecting both SP and Rec in Env-expressing cells, we preferred to employ a Rec expression plasmid [31]. Subcellular localization of HML-2 SP was first examined by biochemical cell fractionation experiments. Following hypotonic lysis, cells were separated by sucrose sedimentation into cytoplasmic, nuclear, nucleoplasmic and nucleolar fractions, and equal relative protein amounts were analyzed by Western blotting. Distribution of the cytoplasmic enzyme GAPDH served as quality control. As shown in Figure 4C, SP and Rec were both found in the cytoplasmic and nuclear fractions. Nuclear fractionation further revealed that HML-2 SP and Rec were predominantly located in the nucleolar fraction. This biochemical analysis was further corroborated by confocal microscopy of EnvΔRec- and Rec-expressing cells. Using the anti-SP antibody, SP and Rec were found enriched in nucleoli. More precisely, co-localization with marker protein B23 showed that SP and Rec, when expressed without tags, located primarily to the granular component of nucleoli (Figure 4D and 4E). Taken together, biochemical and microscopic analyses revealed that HML-2 SP is an additional HML-2 protein produced in HML-2 Env-expressing cells that translocates to the granular component of nucleoli.

### HML-2 SP nucleolar localization is sensitive to inhibition of transcription

**Figure 5:**
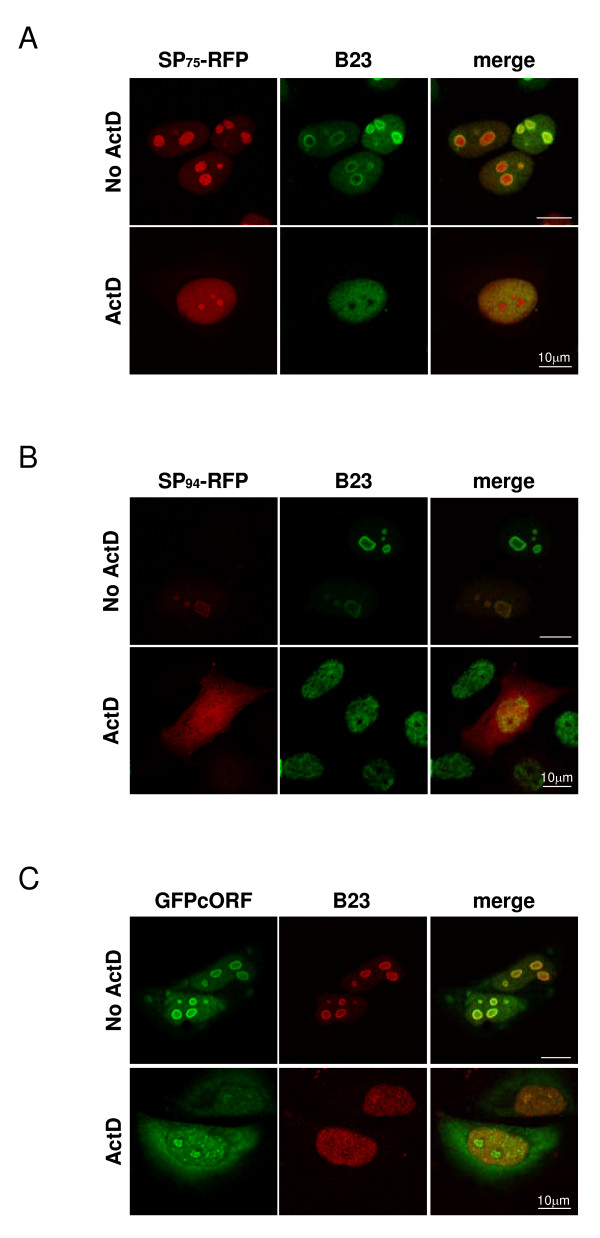
**Effect of actinomycin D treatment on SP distribution**. Confocal analysis of HeLa cells, 24 hours post-transfection, treated (or not) with 5 μg/ml Actinomycin D (ActD/no ActD) for 2 hours. Prior and during the experiment, cells were incubated with CHX at 100 μg/ml. Cells expressing SP_75_-RFP (A), SP_94_-RFP (B) or GFPcORF (C) were fixed and co-immunostained for B23/nucleophosmin nucleoli marker. White bar = 10 μm.

Treatment of cells with Actinomycin D (ActD), an RNA polymerase II inhibitor, causes redistribution of nucleolar proteins, such as B23 and C23, into the nucleoplasm [64,65]. ActD treatment also influences localization of some retroviral proteins. Among those, HIV-1 Rev and HTLV Rex are redistributed to the cytoplasm while MMTV SP translocates from nucleoli to nucleoplasm [64-67]. To further characterize the nucleolar localization of HML-2 SP and Rec proteins we applied 5 μg/ml ActD on SP_75_-RFP, SP_94_-RFP and GFPcORF-expressing cells (Figure 5). Two hours before the start of the experiment, cells were pre-incubated with 100 μg/ml of the protein synthesis inhibitor cycloheximide (CHX) to follow existing SP and Rec protein pools in the absence of new protein production. CHX remained present during the two hours of ActD treatment. Expectedly, addition of ActD caused dispersion of nucleoli markers B23 and C23 from nucleoli to nucleoplasm. SP_75_-RFP also dispersed from nucleoli to nucleoplasm, however, maintained a distinct nucleolar enrichment (Figure 5A). Similarly, GFPcORF inefficiently dispersed from the nucleoli and, in addition, accumulated in the nucleoplasm in a bright punctuate pattern as well as diffusely in the cytoplasm (Figure 5C). Interestingly, SP_94_-RFP completely abandoned the nucleoli and enriched in the nucleoplasm and cytosol (Figure 5B). These results demonstrated that the nucleolar localisation of a precursor protein harboring HML-2 SP is as sensitive to ActD-mediated dispersion as that of cellular markers of granular and dense fibrillar nucleoli components, and revealed further differences in the subcellular localization between Rec and SP fusion proteins upon ActD treatment.

### HML-2 SP is subjected to proteasomal degradation

**Figure 6:**
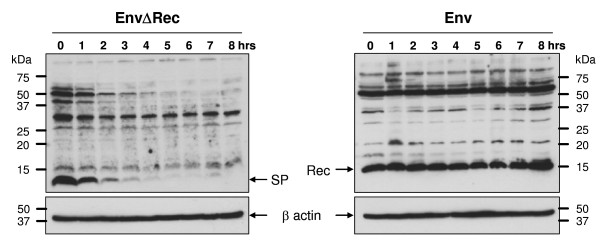
**Determination of half-life of HML-2 SP**. HeLa cells expressing either EnvΔRec or Env were incubated, 16 hours post-transfection, with the translation inhibitor CHX at 100 μg/ml. SP and Rec molecules were chased for up to 8 hours. Every hour, cells were collected and analyzed by Western blot using anti-SP. Cellular β-actin was analyzed as a loading control. Larger-sized protein bands correspond to cytosolic translation products due to saturation effects (see text).

Although HML-2 SP and Rec proteins share large parts of their amino acid sequence and both localize to nucleoli, the steady state protein levels in the context of HML-2 Env expression were significantly different (Figure 4B). To address the reason for these different expression levels we compared the stability of HML-2 SP and Rec proteins. CHX (100 μg/ml) was added to Env- and EnvΔRec-expressing cells 16 hours post-transfection and pre-existing SP and Rec molecules were chased for up to 8 hours. At various time points, cells were collected and analyzed by Western blotting using the anti-SP antibody. β-actin served as loading control. As shown in Figure 6, the amounts of SP present at time point 0 rapidly decreased, resulting in an estimated half-life of the protein of not more than 2 hours. In contrast, cellular Rec levels remained constant over the entire 8 hour chase period. In addition to SP and Rec, we noted higher molecular weight bands in both Env- and EnvΔRec-expressing cells, including a protein band slightly larger than SP. Those proteins most likely resulted from saturation and bypassing of the ER pathway due to large-scale mRNA synthesis and protein production following transient transfection. Translation then initiates on free ribosomes in the cytosol producing polypeptides of variable length that each contain the N-terminal epitope used for anti-SP generation.

**Figure 7:**
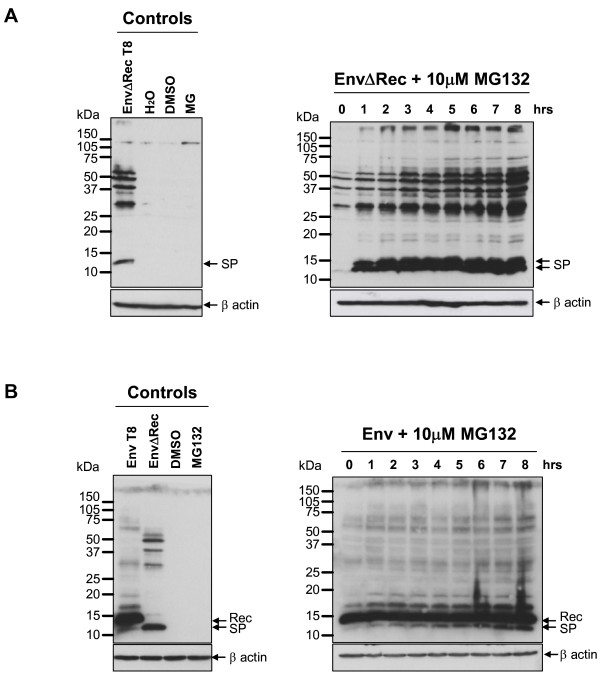
**Degradation of HML-2 SP by the proteasome**. HeLa cells expressing EnvΔRec or Env were incubated, 16 hours post-transfection, with proteasome inhibitor MG132 at 10 μM. Cells were collected every hour for, in total, 8 hours and analyzed by Western blotting using anti-SP. Cellular β-actin was analyzed as a loading control. Larger-sized protein bands, including the protein band slightly larger than SP in the left-hand Western blot, correspond to cytosolic translation products that are due to saturation effects (see text).

To better understand the molecular mechanism responsible for reduced HML-2 SP stability, when compared to Rec, proteasome inhibition was achieved by addition of carbobenzoxy-L-leucyl-L-leucyl-L-leucinal (MG132) to a final concentration of 10 μM. MG132 was added to Env- and EnvΔRec-expressing cells 16 hours post-transfection. Cells were collected every hour and analyzed by Western blot using the anti-SP antibody. As shown in Figure 7, for EnvΔRec-transfected cells, at the start of the incubation and consistent with our previous results, expression of SP was difficult to detect. Treatment with MG132 for one hour drastically increased (at least ten-fold) the amount of SP, and SP levels remained high over 8 hours of incubation in presence of proteasome inhibitor. Above mentioned cytosolic Env-translation products also accumulated in the course of the treatment. To confirm that the difficulty in detecting SP at a steady state in Env-expressing cells was due to its degradation by the proteasome, we applied MG132 to Env-expressing cells. As shown in Figure 7, at the start of the experiment, SP was barely detectable but accumulated after MG132 treatment. In this case, SP levels increased gradually during the 8 hours of treatment. A doublet band of approximatively 14 kDa could also be detected and accumulated in both Env- and EnvΔRec-expressing cells. This observation raised the possibiblity of further processing of HML-2 SP by signal peptide peptidase that is thought to cleave SPs within the hydrophobic transmembrane region. A cleavage in HML-2 SP h domain (residues 76–90) would remove around 12 to 14 aa, corresponding to approximately 1 kDa. Biochemical studies are required to characterize whether HML-2 SP is released in the cytosol after signal peptide peptidase cleavage or extraction from the lipidic membrane as observed recently for p14/SP_Rem _[54]. Altogether, these results reveal that HML-2 SP has a half-life of less than two hours following cleavage from the Env precursor, and is degraded by the proteasome.

### HML-2 SP is not a nucleo-cytoplasmic shuttle protein and lacks RNA export activity

**Figure 8:**
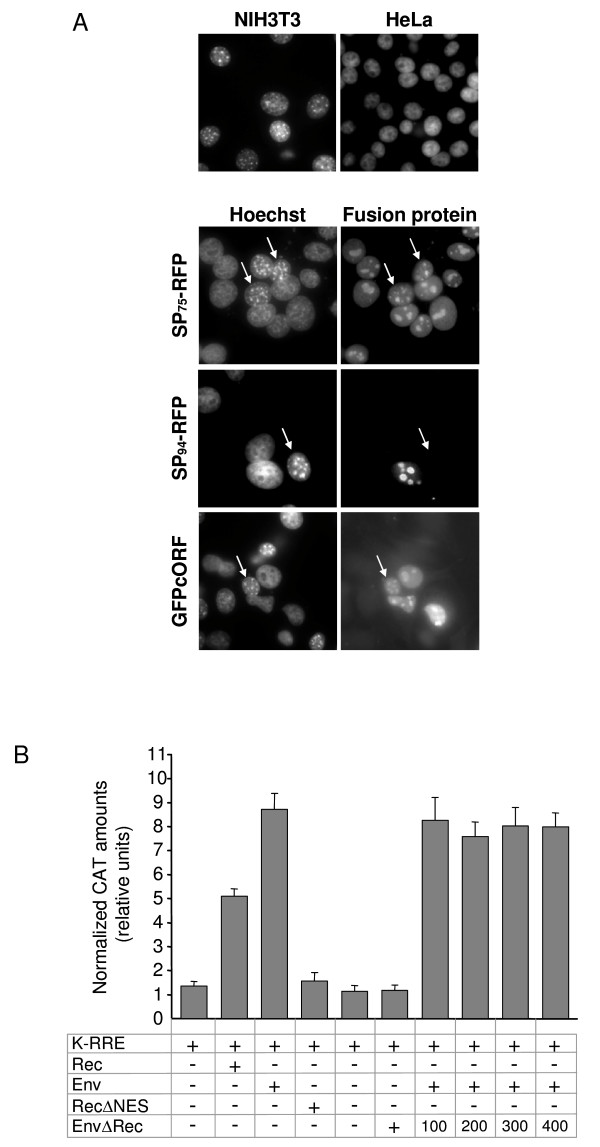
**Functional differences between HML-2 SP and Rec**. (A) Heterokaryon assay. HeLa cells were transfected with SP_75_-RFP, SP_94_-RFP or GFPcORF expression plasmids and co-cultured with mouse NIH3T3 cells. Cells were subsequently treated with 100 μg/ml CHX for 1 hour. After induction of cell fusion, cells were fixed and immunostained. Counterstaining with Hoechst 33258 served to distinguish human from mouse nuclei (left panels). Arrows indicate NIH3T3 nuclei that accumulated SP_94/75_-RFP or GFPcORF after syncytia formation (right panels). (B) Measurement of HML-2 SP RNA export activity by quantitative determination of chloramphenicol acetyltransferase (CAT). HeLa cells were co-transfected with the CAT reporter plasmid pDM128/K-RRE (K-RRE) together with either Rec, Env, RecΔNES, or EnvΔRec expression plasmids. Histogram bars represent normalized amounts of CAT as determined by CAT ELISA, indicating export of unspliced CAT mRNA to the cytoplasm. Where indicated, 400 ng of Env expression plasmid were co-transfected with increasing amounts of EnvΔRec plasmid (100, 200, 300 or 400 ng), and with constant amounts of the CAT reporter vector. All values are given as the mean of at least 4 independent experiments. Error bars indicate the SEM.

The observed differences in protein stability and responsiveness to ActD suggested that HML-2 SP and Rec may have distinct biological properties. Rec's activity in export of genomic RNA strictly depends on functional NLS and NES motifs. While our above analysis demonstrated efficient nuclear import of SP, functionality of the putative NES sequence in SP (see Figure 2) was not addressed. We therefore examined nucleo-cytoplasmic shuttling of SP, compared to Rec, in an interspecies heterokaryon assay [68]. Heterokaryons were formed by fusing transfected human cells with untransfected mouse cells. Mouse nuclei could be distinguished because of a characteristic punctuated pattern following staining with Hoechst 33258 [69] (Figure 8A). HeLa cells were transfected with SP_75_-RFP, SP_94_-RFP or GFPcORF expression plasmids and co-cultured with mouse NIH3T3 cells 16 hours post-transfection. 24 hours later, protein synthesis was blocked by CHX treatment and fusion was induced. Syncytia formation was verified by May-Grünwald-Giemsa staining (not shown). Rec (GFPcORF) was readily detected in human and mouse nuclei, confirming its nucleo-cytoplasmic shuttling activity. SP_75_-RFP also shuttled into mouse nucleoli, even more efficiently than Rec/GFPcORF, demonstrating that the NES motif in SP_75_-RFP is functional. However, SP_94_-RFP was never found in mouse nucleoli, suggesting that in the context of the full-length sequence, the NES motif has lost its functionality. Thus, heterokaryon assays demonstrated that full-length HML-2 SP carries a non functional NES. HML-2 SP therefore does not appear to be a nucleo-cytoplasmic shuttle protein.

We also asked whether HML-2 SP could export HML-2 mRNA in a manner similar to Rec. To export HML-2 transcripts to the cytoplasm, Rec specifically binds to the Rec-responsive element, K-RRE, located within the U3 region of the HML-2 long terminal repeat [28,29]. To quantify RNA export activity, HeLa cells were transfected with the chloramphenicol acetyl transferase (CAT) reporter plasmid pDM128/K-RRE [29]. Cytoplasmic expression of CAT protein was measured in response to co-expressed Rec and EnvΔRec. pDM128/K-RRE carries the *cat *gene within an intron under control of the SV40 promoter flanked downstream by the K-RRE sequence [29]. Presence of a K-RRE-binding RNA export factor thus leads to export of unspliced CAT mRNA to the cytoplasm and CAT protein synthesis. 24 hours post-transfection, the cytoplasmic levels of CAT were determined by CAT ELISA. As shown in Figure 8B, co-transfection of pDM128/K-RRE and expression plasmids for Rec alone, or for HML-2 Env, resulted in a 4- and 6-fold, respectively, increase of CAT expression over background. As expected, an RNA export-defective Rec mutant (RecΔNES), carrying a mutated NES motif [70] displayed no significant RNA export activity. We next tested HML-2 SP's export activity by co-transfecting the reporter plasmid along with EnvΔRec expression plasmids. No significant induction of CAT production was detected for SP. We thus conclude that HML-2 SP, in contrast to Rec, lacks HML-2 mRNA export activity.

Since SP and Rec both reside in nucleoli, and since SP is highly similar in sequence to Rec, we also addressed whether HML-2 SP can affect Rec's RNA export activity. However, co-expression of Env with increasing concentrations of EnvΔRec, together with constant amounts of the reporter vector, had no effect on CAT levels (Figure 8B). These results suggest that even if SP accumulates in the nucleoli, it does not sequester HML-2 mRNA away from Rec and thus can not compete with Rec for mRNA binding. This indicates that, despite its high sequence similarity to Rec, SP exerts (a) function(s) distinct from Rec.

## Discussion

This study demonstrates a previously unrecognized protein encoded by HERV-K(HML-2), namely a 13 kDa SP that is produced by cleavage of the HML-2 Env precursor. Sequence analysis revealed that HML-2 SP is common to HERV-K(HML-2) type 2 proviruses and that the first 87 residues, out of 96, are shared with Rec, the protein exporting HERV-K(HML-2) mRNA out of the nucleus [27-29]. Like Rec, HML-2 SP is found in the granular component of nucleoli. In contrast to Rec, HML-2 SP is rather short-lived, it is subjected to proteasomal degradation, it does not shuttle between nucleoli and cytosol, and it does not export HML-2 mRNA out of the nucleus. These observations reveal that HML-2 type 2 proviruses encode an additional Rec-like protein that is functionally distinct from Rec. Despite its proteasomal degradation, HERV-K(HML-2) SP is a long-lived protein when compared to conventional SPs. Thus, in addition to its primary function of targeting the Env precursor to the secretory pathway, HML-2 SP traffics to nucleoli after its cleavage from the Env precursor to exert an independent, yet to be characterized activity.

Considering that HML-2 SP shares 87 of its 96 residues with Rec, the functional differences between both proteins are remarkable. These differences do not stem from altered subcellular localization. Surprisingly, while the nuclear import motif is functional in HML-2 SP, mediating its transport into the nucleoli, the NES motif, functional and well characterized for Rec, is no longer functional in HML-2 SP. Shuttle activity, as well as distinct protein stability, and RNA export inactivity are therefore probably determined by the HML-2 C-terminal 9 aa that distinguish SP from Rec. One may argue that reduced half-life of HML-2 SP, when compared to Rec, hampers its ability to export HML-2 RNA. Addition of ubiquitin on protein chains is known to target them to the proteasome [71,72]. SUMOplot [73] predicts a putative sumoylation motif between residues 37 and 40 (MKLP) of both Rec and HML-2 SP. One might hypothesize that this region is more accessible in SP than in Rec due to differences in the 3D conformation of both proteins. Alternatively, the unique C-terminus of HML-2 SP may facilitate interaction with e.g. the ubiqutin ligase system. However, if decreased protein stability was the limiting factor, HML-2 SP's RNA export activity would be reduced rather than absent. The observed nonfunctional NES motif, in combination with a complete lack of RNA export activity, thus suggests general impairment of HML-2 SP in RNA transport.

Signal peptide fragments resulting from processing of signal peptides by signal peptide peptidases are known to be released in the cytosol. Such processing could also occur in the hydrophobic region of HML-2 SP. Recent biochemical analysis of p14/SP_Rem_, also trafficking to nucleoli after its cleavage, showed that its release was independent of Signal Peptide Peptidase activity. The release was a time- and temperature-dependent process, and SP_Rem_/p14 was found in the nucleolar fraction for at least 90 minutes [54]. It is currently not known whether MMTV p14/SP_Rem _and HML-2 SP are processed in the same manner.

The fact that SP sequences are common to HML-2 type 2 proviruses argues for a vital role of SP in the former viral life cycle. The presented data rule out that HML-2 SP served to back up or modulate Rec's main function – RNA export activity. SPs of other viral proteins also exert functions beyond ER targeting during the viral life cycle. In cases where specific functions could be attributed to the viral SP, most of these activities relate to the maturation and function of the respective viral glycoprotein and affect processes such as Env folding or post-translational modifications (HIV-1 gp120 and Ebola SP) [51,74-76], complex formation with the mature Env glycoprotein (Junin Virus and Lymphocytic Choriomeningitis Virus), intracellular trafficking, fusion or virus infectivity in general [77-80]. In HFV, SP affects viral particle assembly and release [53,81]. When testing potential effects of HML-2 SP on Env expression, we did not observe post-translational impairment, as HML-2 Env precursor was efficiently cleaved in the Golgi into SU and TM subunits, was glycosylated on 8 potential N-glycosylation consensus sites and was found expressed on the cell surface of human transfected cells (A. Ruggieri, unpublished results). HML-2 SP therefore does not seem to regulate or delay Env complex maturation or cell surface expression. However, due to the lack of infectious HERV-K(HML-2) viral particles one cannot exclude that HML-2 SP affects Env's fusion functions.

The data presented thus far do not allow us to conclude of what the former and potentially current biological activity of HML-2 SP consists. The same is true for a comparison of HML-2 SP functions with those of the related MMTV p14/SP_Rem_, especially when considering that both have not been characterized in great detail, yet. In the context of human biology, when assuming a causal relationship between expression of HML-2 gene products and germ cell tumor development, active immune evasion mechanisms are likely in place to counteract elimination of HML-2 protein-expressing cells. Tumor cells and virally infected cells often display significantly reduced human leukocyte antigen (HLA) cell surface levels, rendering them prone to attack and lysis by natural killer (NK) cells. Viruses and tumor cells have therefore evolved various strategies to interfere with NK cell recognition and activation [82]. Of note, a key cellular mechanism for evasion of NK cell lysis involves SPs. SP fragments from cellular proteins containing long and hydrophilic N-extension can be presented by HLA molecules following release into the cytosol, proteasomal degradation, transport into the ER lumen by the transporter of antigen presentation, and subsequent presentation to the cell surface [83,84]. Antigen-presenting cells make use of this mechanism to report biosynthesis of MHC class I molecules to the immune system [85]: following proteasomal degradation, SPs of HLA-A, B and C molecules bind selectively to HLA-E molecules that present them to the inhibitory receptor (CD94-NKG2) on NK cells, thereby preventing lysis by NK cells. Remarkably, human cytomegalovirus (HCMV) makes use of that escape route via a 9aa signal peptide-derived fragment from the HCMV UL40 glycoprotein [86]. Worth mentioning, a Rhesus CMV-encoded protein was shown to interfere, in a signal sequence-dependent manner, with the MHC-I pathway by preventing completion of heavy chain translation [87]. Based on its short half-life, due to the action of the proteasome, one might speculate that HML-2 SP peptides also re-enter the ER lumen to be presented by HLA molecules, e.g. by HLA-E to interfere with NK lysis of HERV-K(HML-2) expressing cells. Thus, HML-2 SP could be involved in the evasion of GCT cells from the immune system. Alternatively, HML-2 SP may interact with other cellular proteins in, for instance, nucleoli to interfere with cellular processes. Interestingly, MMTV p14/SP_Rem _has been reported to interact with B23/nucleophosmin [39] that has been attributed with tumor suppresor functions [88]. HML-2 SP may likewise interact with B23/nucleophosmin and may thus interfere with important cellular processes. Building on our initial detection and characterization of HML-2 SP, future studies will have to elucidate the mechanism of action of HML-2 SP and HML-2 SP's potential significance for GCT.

## Conclusion

The human endogenous retrovirus family HERV-K(HML-2) is exceptional for various reasons, among them its protein coding capacity. We now found that the HERV-K(HML-2) *env *gene gives rise to an additional 13 kDa protein by cleavage of the signal peptide from the Env precursor. The HML-2 SP repesents another functional similarity with the related exogenous Mouse Mammary Tumor Virus that likewise encodes a stable signal peptide, adding another case to the concept of several retroviral SPs having biological functions besides ER-targeting. HML-2 SP and HML-2 Rec protein, the latter potentially involved in germ cell tumorigenesis, are very similar in sequence, except for the N-terminus. Our results strongly indicate that HML-2 SP lacks several functional features previously reported for HML-2 Rec. HML-2 SP is therefore expected to exert a different biological function. Besides a distinct function in the (former) retroviral context, HML-2 SP must also be considered in the context of human biology. Given the biological features found for HML-2 SP, and findings for the MMTV SP, one can hypothesize that HML-2 SP is involved in immune evasion of GCT cells. HML-2 SP may also interact with important cellular proteins, thereby playing a significant role in GCT development. Future research must address HML-2 SP in greater detail.

## Methods

### Cell lines, reagents and expression plasmids

HeLa and NIH3T3 cells were maintained in Dubelcco's modified Eagle medium (Invitrogen) supplemented with 10% fetal calf serum (Biochrom). phCMV-HML2-Env was derived from phCMV-G, a VSV-G Env expression plasmid [89] by replacing VSV-G Env sequence with HML-2 Env sequence. HML-2 env gene was amplified by PCR from the HERV-K(HML-2.HOM) locus (GenBank accession no. AF074086) [60] and inserted between the two *Eco*RI sites of phCMV-G. pSP_75_-RFP, pSP_94_-RFP and pSP_96_-RFP plasmids express respectively the N-extension region (aa 1–75), the sequence missing the last two aa (aa 1–94) and the full length sequence (aa 1–96) of HML-2 signal peptide (SP), each fused to monomeric Red Fluorescent Protein (mRFP). SP sequences were amplified by PCR from phCMV-HML2-Env and inserted into the *Ecl*136II site of pmRFP-N1 multiple cloning site [90]. phCMV-EnvΔRec, an Env expression vector deficient for splicing of *rec *mRNA, was generated by site-directed mutatgenesis (QuickChange Site-Directed Mutagenesis Kit, Stratagene) of Rec splice donor and acceptor sites located at aa 85–87 and aa 651–655, respectively, of Env. Integrity of generated plasmids was verified by sequencing. Expression plasmids for HML-2 Rec, pEGFP-wtcORF and pSG5-Rec, were described elsewhere [31,61]. pDM128/K-RRE and pK7-K-RevDNES vectors [29,70] were kindly provided by Bryan Cullen. pEGFP-N1 (Clontech) was used as control for transfection efficiency and for normalization in the CAT assay.

### Antibodies

A rabbit anti-SP antibody was raised against HML-2 Env signal peptide residues 1–19 (MNPSEMQRKAPPRRRRHRN) and was used unpurified at a 1:1,000 dilution for Western blot analysis and 1:200 for immunofluorescence. The following antibodies were used at the indicated concentration: mouse monoclonal anti-nucleophosmin B23 (Zymed Laboratories, Invitrogen) at a 1:1,000 dilution for Western blotting and 1:50 for immunofluorescence; mouse monoclonal anti-nucleolin C23 (clone MS-3) (Santa Cruz Biotechnology) at a 1:50 dilution for immunofluorescence; mouse monoclonal anti-GAPDH (clone 0411) (Santa Cruz Biotechnology) at a 1:200 dilution for Western blot analysis; mouse monoclonal anti-β actin (clone AC-15) (Sigma) at a 1:10,000 dilution for Western blotting. Horseradish peroxidase-conjugated secondary antibodies were purchased from Jackson ImmunoResearch Laboratories (Dianova): goat anti-rabbit IgG (dilution 1:20,000) and sheep anti-mouse IgG (dilution 1:30,000). Fluorescent secondary antibodies were purchased from Molecular Probes (Invitrogen) and used at a 1:2,000 dilution: Alexa Fluor 488 goat anti-mouse IgG, Alexa Fluor 568 goat anti-mouse IgG, and Alexa Fluor 568 goat anti-rabbit IgG.

### Western blot analysis

Transfected cells were harvested 48 hours post-transfection and lyzed in cold NP40 lysis buffer (50 mM Tris-HCl pH8, 150 mM NaCl, 1% NP40) supplemented with protease inhibitor cocktail (Complete Mini EDTA free, Roche). Samples were boiled in 5× Laemmli sample buffer, separated by 15% SDS-PAGE, and transferred onto Hybond-P membrane (Amersham). Membranes were blocked with Tris Buffered Saline containing 5% skim milk and 0.05% Tween 20 (Sigma) overnight at 4°C. Immunostaining was performed in the same buffer using appropriate first and secondary antibodies. Protein detection was performed by using the ECL Plus detection kit (Amersham) according to the manufacturer's instructions.

### Cell fractionation

Cell fractionation was adapted from a protocol by Lam and Lamond [91] with modifications as described. Thirty hours after transfection, cells were detached and washed in pre-warmed PBS. All steps were performed on ice. Cells were resuspended in RBS-8 buffer (10 mM Tris-HCl pH7.5, 10 mM NaCl, 8 mM Mg acetate) and incubated on ice for 30 min. Cells were centrifuged (220 g, 5 min, 4°C), resuspended in 500 μl RBS-NP40 lysis buffer (10 mM Tris-HCl pH7.5, 10 mM NaCl, 1.5 mM Mg-acetate, 0.5% NP40) containing protease inhibitor cocktail and were then transferred into a pre-cooled Dounce tissue homogenizer. After 30 to 50 strokes, the cell suspension was centrifuged (220 g, 5 min, 4°C) to separate the cytoplasmic fraction from the nuclear pellet. The cytoplasmic fraction was further centrifuged (5,000 g, 3 × 10 min, 4°C) to eliminate cell debris. To further purify nuclei, the nuclear pellet was washed three times in 3 ml of S1 solution (0.25 M sucrose, 10 mM MgCl_2_, protease inhibitor cocktail). After the final wash, the nuclear pellet was resuspended in 3 ml of S1 solution and layered over 3 ml of S2 solution (0.35 M sucrose, 0.5 mM MgCl_2_, protease inhibitor cocktail). The sucrose cushion was centrifuged (1,430 g, 5 min, 4°C). The purified nuclear pellet was resuspended in 500 μl of S2 solution and sonicated (3 × 20 sec on ice). The sonicated nuclei suspension was layered over 1 ml of S3 solution (0.88 M sucrose, 0.5 mM MgCl_2_, protease inhibitor cocktail) and centrifuged (3,000 g, 30 min, 4°C). The upper fraction was collected and used as nucleoplasm fraction; the pellet contained the purified nucleoli. All extracts were adjusted with 2× Laemmli sample buffer, sonicated and boiled before measuring protein concentration.

### Immunofluorescence

To assay the subcellular localisation of the signal peptide, 2.5 × 10^5 ^HeLa cells were seeded on glass coverslips and transfected with 2 μg expression plasmids. Twenty-four hours after transfection, cells were fixed with 4% paraformaldehyde in Phosphate Buffered Saline (PBS) for 20 min and permeabilised with 0.1% Triton X-100 in PBS for 1 min. Cells were washed once with PBS before being treated with blocking solution (1% goat serum in PBS) for 15 min. All incubations were done at room temperature; washing steps consisted of three PBS washes of 5 min each. Cells were incubated with rabbit anti-SP, anti-nucleophosmin B23, anti-nucleolin C23 and appropriate fluorescent secondary antibodies. Finally, coverslips were mounted using Histoprime (Histogel, Linaris). Fluorescent microscopy images were acquired using an LSM 510 confocal laser scanning microscope (Zeiss) and using a 60× oil immersion objective lens. Confocal images were processed using Adobe Photoshop (Adobe Systems).

### Determination of protein half-life

1 × 10^6 ^HeLa cells were grown in 60 mm tissue culture plates. 16 hours post-transfection, cycloheximide (CHX, Sigma) was added to a final concentration of 100 μg/ml to terminate protein synthesis. Cells were then collected every hour until 8 hours later. Cell lysates were prepared as described above and analyzed by Western blot.

### Proteasome inhibition

1 × 10^6 ^HeLa cells were grown in 60 mm tissue culture plates. 16 hours post-transfection, cells were incubated for 8 hours in the presence of 10 mM Carbobenzoxy-L-Leucyl-L-Leucyl-L-Leucinal (MG132, Calbiochem) diluted in dimethyl sulfoxyde (DMSO, Sigma). Mock samples and DMSO-treated cells were co-cultured in parallel as controls. Cell lysates were prepared as described above and analyzed by Western blot.

### Heterokaryon assay

2.5 × 10^5 ^HeLa cells were transfected with 2 μg pGFPcwtORF, pSP_75_-RFP or pSP_94_-RFP expression plasmids. Twenty-four hours after transfection, cells were mixed with 1 × 10^6 ^NIH3T3 cells and seeded onto 1.8 × 1.8 cm glass coverslips. Twelve hours later, cells were treated with 100 μg/ml CHX for 1 hour, and fusion of cell membranes was induced by addition of 50% (w/v) polyethylene glycol 8000 (Sigma) in DMEM for 2 min at 37°C. Cells were subsequently washed three times with pre-warmed PBS and once with DMEM, before being incubated at 37°C for another 6 hours in the presence of 100 μg/ml CHX. Finally, cells were fixed for immunostaining as described above. Human and mouse nuclei were distinguished by staining with Hoechst 33258 (Sigma) at 0.5 μg/ml in PBS. Fluorescent microscopy images were acquired using an LSM 510 microscope (Zeiss). Images were processed using Adobe Photoshop (Adobe Systems).

### CAT ELISA assay

The mRNA export function of the HML-2 signal peptide was assessed by quantitative determination of chloramphenicol acetyltransferase (CAT) in a CAT ELISA assay. 5 × 10^5 ^HeLa cells were co-transfected with the CAT reporter plasmid pDM128/K-RRE and either pSG5-Rec, phCMV-HML2-Env, pK7-K-RevΔNES (RecΔNES) or phCMV-EnvΔRec, and pEGFP-N1 as a control for transfection efficiency. The CAT ELISA Kit (Roche) was used according to the manufacturer's instructions. Briefly, 24 hours after transfection, cells were lyzed and CAT-containing cell extracts were added into microplate wells coated with anti-CAT antibody, followed by addition of a digoxigenin-labeled anti-CAT antibody, and by addition of a peroxidase-conjugated anti-digoxigenin antibody. After addition of peroxidase substrate, the absorbance of the colored reaction product was measured at 405 nm (reference wavelength 490 nm) by using a microplate reader ELX-800 (Bio-Tek Instruments). Observed CAT production was normalized by using GFP as an internal control. GFP fluorescence of individual cells was analyzed by flow cytometry using a FACS Scan (Becton Dickinson).

## Competing interests

The authors declare that they have no competing interests.

## Authors' contributions

AR, EM, MS and OF planned and performed experiments. AR, OF and JM conceived the study. NML, EM, OF and JM financed the study. AR, OF and JM wrote the paper. All authors read and approved the final manuscript.
